# Endoscopic submucosal dissection of a duodenal laterally spreading tumor using saline immersion therapeutic endoscopy

**DOI:** 10.1055/a-2589-0996

**Published:** 2025-05-19

**Authors:** Shaimaa Elkholy, Abeer Abdallatef, Mohamed Abdel Zaher, Karim Essam, Hany Haggag, Kerolis Yousef, Mohamed El-Sherbiny

**Affiliations:** 1 63527 Gastroenterology Division, Internal Medicine Department, Faculty of Medicine, Cairo University Kasr Alainy, Cairo, Egypt; 2 63527 MBBCh, Faculty of Medicine, Cairo University Kasr Alainy, Cairo, Egypt

ESD is an advanced endoscopic procedure that enables therapeutic en bloc resection of gastrointestinal lesions. Because of the variability in lesion characteristics and anatomical challenges, each case requires a tailored approach 1 .

In recent years, saline immersion therapeutic endoscopy (SITE) has gained prominence as an effective adjunct in ESD. SITE enhances visibility by eliminating the air-liquid interface, allowing for precise identification of the submucosal layer, which is critical for a safe and successful dissection 2 3 . The buoyancy effect stabilizes the lesion, reducing the need for additional traction devices and improving scope maneuverability, thus increasing procedural safety 4 5 . Moreover, continuous saline flushing maintains submucosal lift providing a stable dissection field 3 5 . SITE also helps in reducing the gas-related complications.

We present a case of a 65-year-old man discovered to have a laterally spreading tumor, measuring 40 × 30 mm, at the junction between the second and third parts of the duodenum ( Fig. 1 ). Given the thin-walled duodenum, the procedure was challenging. After consultation with a multidisciplinary team, ESD using the SITE technique was planned. Continuous saline flushing was performed using the scope’s water pump ( Video 1 ). The pocket creation method was employed, starting with a mucosal incision from the oral side of the lesion to create a short submucosal tunnel, followed by the completion of the circular incision. SITE facilitated clear delineation of the submucosal structures (Video Image).

**Fig. 1:**
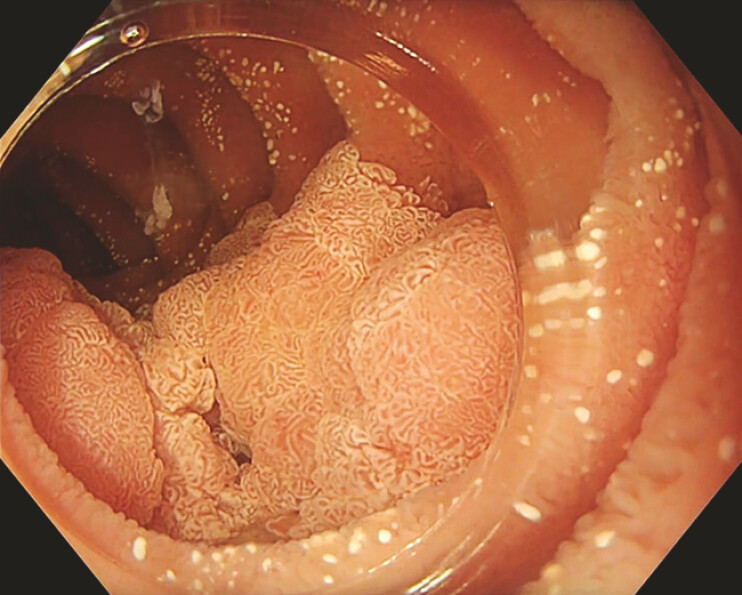
Duodenal laterally spreading tumor.

Demonstration of endoscopic submucosal dissection of a duodenal laterally spreading tumor using saline immersion therapeutic endoscopy (SITE).Video 1

En bloc resection was successfully achieved ( Fig. 2 ), and after proper inspection of the lesion bed, it was closed with hemoclips ( Fig. 3 ). Histopathology confirmed R0 resection of a tubulovillous adenoma with high-grade dysplasia and clear margins ( Fig. 4 , Fig. 5 ). The procedure was performed using an Olympus scope ×1 (1500), a FINEMEDIX knife (CO, LTD) with a 1.5 mm tip, and ERBE Vio3 (Endocut I 2,3,2; swift coagulation 2.5) ( Video 1 ).

**Fig. 2:**
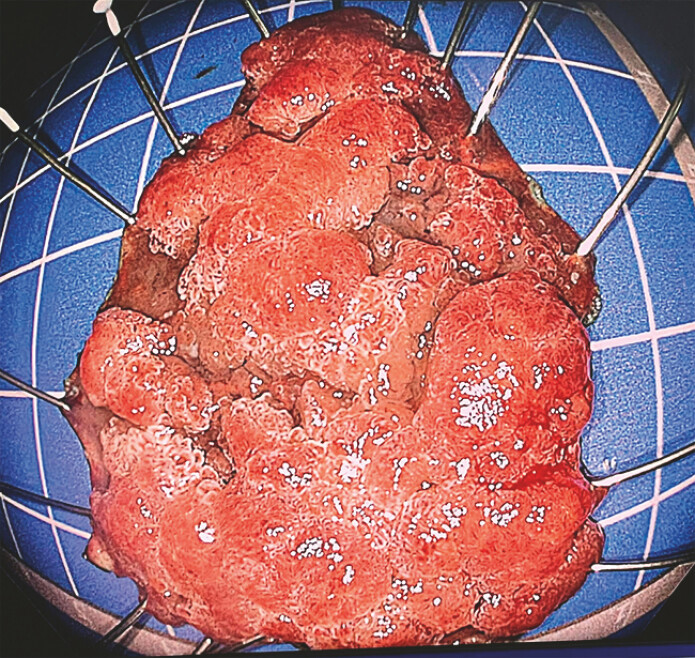
Retrieved lesion after en bloc resection.

**Fig. 3:**
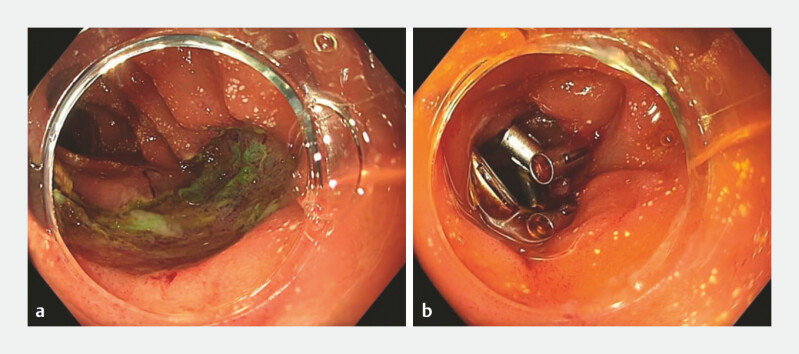
**a** Resection bed, **b** bed closure with hemoclips.

**Fig. 4:**
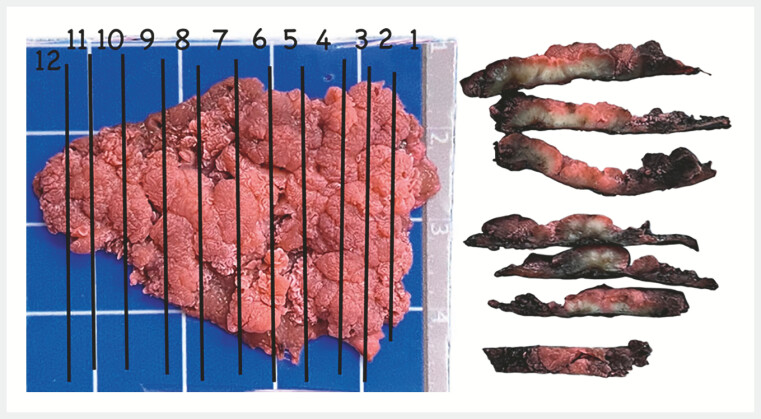
Gross mapping.

**Fig. 5:**
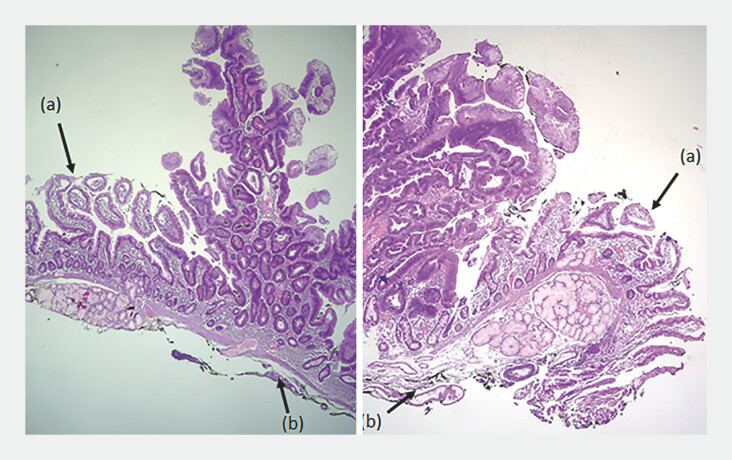
Histopathology confirms tubulovillous adenoma with high-grade dysplasia. Free lateral margins (a), free deep margins (b).

Endoscopy_UCTN_Code_TTT_1AO_2AG_3AZ

## Notes

Endoscopy E-Videos https://eref.thieme.de/e-videos
*E-Videos* is an open access online section of the journal *Endoscopy* , reporting on interesting cases and new techniques in gastroenterological endoscopy. All papers include a high-quality video and are published with a Creative Commons CC-BY license. Endoscopy E-Videos qualify for HINARI discounts and waivers and eligibility is automatically checked during the submission process. We grant 100% waivers to articles whose corresponding authors are based in Group A countries and 50% waivers to those who are based in Group B countries as classified by Research4Life (see: https://www.research4life.org/access/eligibility/ ). This section has its own submission website at https://mc.manuscriptcentral.com/e-videos .
